# Investing in climate change adaptation and mitigation: A methodological review of real-options studies

**DOI:** 10.1007/s13280-020-01342-8

**Published:** 2020-05-25

**Authors:** Tsegaye Ginbo, Luca Di Corato, Ruben Hoffmann

**Affiliations:** 1 Department of Economics, Swedish University of Agricultural Sciences, Box 7013, 75007 Uppsala, Sweden; 2 Department of Economics, Ca’ Foscari University of Venice, Cannaregio 873, Fondamenta San Giobbe, 30121 Venice, Italy

**Keywords:** Climate change adaptation, Climate change mitigation, Real-options, Uncertainty

## Abstract

Uncertain future payoffs and irreversible costs characterize investment in climate change adaptation and mitigation. Under these conditions, it is relevant to analyze investment decisions in a real options framework, as this approach takes into account the economic value associated with investment time flexibility. In this paper, we provide an overview of the literature adopting a real option approach to analyze investment in climate change adaptation and mitigation, and examine how the uncertain impacts of climate change on the condition of the human environment, risk preferences, and strategic interactions among decisions-makers have been modeled. We found that the complex nature of uncertainties associated with climate change is typically only partially taken into account and that the analysis is usually limited to decisions taken by individual risk neutral profit maximizers. Our findings call for further research to fill the identified gaps.

**Electronic supplementary material:**

The online version of this article (10.1007/s13280-020-01342-8) contains supplementary material, which is available to authorized users.

## Introduction

It is widely recognized that humanity needs to take action to limit and reduce risks associated with climate change (IPCC 2014a, b, c, d, e). Actions required to deal with the risks associated with climate change include (i) adaptation, i.e. effective adjustments to actual or expected climatic shocks and their effects to increase resilience and reduce vulnerability (IPCC 2014a) and (ii) mitigation, i.e. measures aimed at reducing GHG emissions (IPCC 2014c, d). There have been several global initiatives aimed at tackling and limiting the impact of climate change, e.g. the Kyoto Protocol and the Paris Agreement.

Although these initiatives have resulted in agreements on specific targets, e.g. on the emissions of carbon dioxide, actions to achieve these targets have been limited and, consequently, have not been reached (OECD 2018). For example, total GHG emissions have, contrary to agreed targets, increased between 2000 and 2010 (IPCC 2014d, e). The actions undertaken so far are insufficient to keep the increase in average surface temperature below 2 °C, as agreed at the COP 21 in Paris (OECD 2018). Substantial uncertainties concerning the future climate and the potential impact of alternative actions may explain why observed mitigation and adaptation activities are limited (Heal and Millner 2014; IPCC 2014e). Furthermore, many required investments are costly and irreversible. Uncertainty, in combination with irreversible investments, provides incentives to delay investments since waiting is valuable as it allows for the acquisition of information on future prospects (see e.g., Schou et al. 2015; Chesney et al. 2017; Hauer et al. 2017).

The optimal timing and magnitude of actions are crucial when it comes to investment strategies coping with climate change under uncertainty, as illustrated by Watkiss et al. (2015), Abadie et al. (2017) and Chesney et al. (2017). The traditional Net Present Value (NPV, hereafter) criterion is problematic since it does not account for the value of the option to wait, a value that is implicitly lost once an investment is made.^1^ However, this limitation can be overcome by adopting a real-options approach. In this framework, the decision-maker can delay investments and learn from new information and/or, once the investment has been made, handle other valuable options such as the options to adjust, expand or abandon a specific investment project (Trigeorgis and Reuer 2017). Consequently, scholars have widely adopted the real-options approach in analyzing investments in climate change adaptation and mitigation. In this respect, excellent reviews are provided by Yousefpour et al. (2012) in the context of adaptive forest management and by Golub et al. (2014), Watkiss et al. (2015) and Dittrich et al. (2016) comparing the real-options approach with other approaches.

However, as far as we know, no review exists that focuses on the use of the real-options approach in the general context of investment in climate change adaptation and mitigation, a gap that has motivated our paper. In this paper, we present a systematic review of studies adopting the real-options approach in analyzing investment in climate change adaptation and mitigation. The objectives of this paper are to provide an overview of this literature and to examine how these studies (i) have modeled the problem (ii) have taken the uncertain impacts of climate change on the condition of the human environment into account, (iii) have incorporated risk preferences and (iv) have taken the strategic interactions between the concerned agents into account. In the following sections, we describe the methodology adopted for the review and then present the elements characterizing the decision context and the real-options approach. Subsequently, we present the review of the literature and conclude with a discussion of potential implications for future research.

## Review methodology

We adopted a systematic review methodology in line with the Preferred Reporting Items for Systematic Reviews and Meta-Analyses (PRISMA) as described in Moher et al. (2009) and Moher et al. (2015). In addition, we follow the procedure suggested by Webster and Watson (2002) to track citations backward and forward in order to identify potentially relevant papers not captured in the initial search.

In the systematic literature review, we searched the Scopus, Google Scholar, Web of Sciences and EconLit databases. Articles published between 1973 and 2018 were included. The starting year was chosen because the seminal paper by Black and Scholes (1973), fundamental to the development of the theory of option pricing, was published then. In order to identify papers adopting a real-options approach to the analysis of investment in climate change adaptation and mitigation, we used the search strings (terms) “climat* change adaptation”, “climat* change resilien*”, “climat* change mitigation”^2^ and, “GHG emission reduction”. Each of these strings was combined with the string “real options” using the ‘AND’ operator and the resulting sets were combined altogether using the ‘OR’ operator. This procedure resulted in a set of 716 papers, 285 of which remained after duplicates (308), literature reviews, book chapters and unpublished papers (123), were excluded.

In a second step, papers that did not meet the following criteria were excluded: (a) published as a peer-reviewed article, (b) written in English, (c) primarily focused on investments in climate change adaptation or mitigation, and (d) explicitly applied a real-options analysis. Using these criteria, we identified 58 relevant papers. In these papers, citations were tracked backward and forward allowing us to identify 9 additional papers which, together with the 58 papers previously identified, led to the final set of 67 articles considered in our review.

Finally, for analytical purposes, we constructed a table^3^ where, for each of the selected papers, we extracted and compiled information about (1) the context, specifically the focus of the paper and type of strategies examined, (2) the methodology, specifically the unit of analysis, underlying assumptions, stochastic processes assumed, types of uncertainties included in the models, consideration of strategic interactions, and solution methods, and (3) the main results. The analysis of the literature was conducted based on the extracted information.

## Climate policy and real-options analysis

### Climate change adaptation and mitigation decisions

Actions to cope with climate change involve choosing effective adaptation and mitigation strategies. Climate adaptation aims to (i) avoid or minimize the harmful effects of climate change and (ii) benefit from the potential opportunities associated with climate change (IPCC 2014a). Common examples of adaptation strategies include investments in flood risk control, introduction of new crop varieties, investment in more efficient irrigation and resource-saving technologies, adoption of sustainable forest management, investment in early warning and information sharing systems, soil and water conservation, livelihood diversification, and insurance (IPCC 2014a, b). Climate change mitigation, on the other hand, concerns sectoral and cross-sectoral interventions reducing and/or offsetting emissions of GHGs (IPCC 2014d, e). These include decarbonization of electricity generation, adoption of clean energy technologies, investment in technologies that capture and store carbon dioxide, afforestation, reduction of deforestation, improving grazing land management, and bioenergy production (IPCC 2014c, d). In addition, incentivizing changes in behavior and lifestyles may also be important to reduce GHG emissions (IPCC 2014e).

Both adaptation and mitigation actions require making decisions in an uncertain environment. Climate change involves both scientific and socio-economic uncertainty and the way these uncertainties are taken into account is crucial to the formulation of successful climate policy (Heal and Millner 2014). Scientific uncertainty refers to the uncertainty about the global climate’s sensitivity to changes in the atmospheric composition. Socioeconomic uncertainty concerns the difficulty in forecasting the impacts of climate change and the ways societies may react to climate change (Quiggin 2008; Heal and Millner 2014). In addition, further complicating decision-making, the uncertainty characterizing climate change is, in some respects, deep and dynamic (Refsgaard et al. 2007; Buurman and Babovic 2016). It is deep because the likelihood of an unknown climatic event is highly uncertain, and it is dynamic as this uncertainty may evolve over time. This means that decision-makers can increase their knowledge regarding climate change and its impact through learning over-time (Quiggin 2008; Dittrich et al. 2016; Erfani et al. 2018). Flexibility concerning the timing of action is thus essential.

### Option value and real-options analysis

Irreversibility and option value have, starting with Fisher and Krutilla (1974), been extensively discussed in the environmental economics literature. There are two relevant and partly conflicting types of irreversibility in the context of climate change. Environmental irreversibility refers to the irreversible accumulation of GHGs in the atmosphere (Ha-Duong 1998; Mäler and Fisher 2005; Sims and Finnoff 2016). This suggests that policies limiting emissions should be promoted before it gets to a tipping point beyond which the environmental damage becomes irreversible. On the other hand, investment irreversibility refers to fixed investment costs that, once undertaken, cannot be recovered. This suggests that it may be valuable to delay actions until more information becomes available. This value of waiting is what in the literature is referred to as an option value. The value of learning from the revealed state of nature and from past actions in a sequential decision process have been highlighted in several studies (see e.g., Nordhaus 1994; Ha-Duong 1998). While investment irreversibility suggests that there is an option value associated with waiting before investing in mitigation and adaptation (see e.g., Ha-Duong 1998; Wesseler and Zhao 2019), environmental irreversibility suggests there may be considerable costs associated with postponing such actions.

In the light of this trade-off, the main challenge is specifying the timing and magnitude of actions, a challenge that can be properly handled within a real-options framework. Within this framework, the alternative actions is to invest “now or later” rather than, as in the standard NPV approach, invest “now or never”. This difference is crucial in that it allows taking into account not only “whether” but also “when” an investment should be undertaken. In other words, it allows taking into account the role managerial flexibility may play in reducing potential losses in an investment context characterized by uncertainty and irreversibility.

### Modelling uncertainty

Real-options models in the context of climate change typically capture uncertainty by considering different scenarios for the future climate and stochastic benefits and/or costs associated with the investment. Typically, the stochastic process of a variable of interest is assumed to follow a Geometric Brownian motion (GBM, hereafter), an Arithmetic Brownian Motion (ABM, hereafter) or a Mean-Reverting process (MR, hereafter). A GBM is used when the stochastic variable may take only positive values and is log-normally distributed, whereas an ABM is appropriate for variables which may take also negative values and have a normal distribution. A MR process is used when variables are expected to evolve around a long-run mean. Finally, to further characterize the evolution of the variable of interest, one may allow for jumps driven by a Poisson process which can capture the impact of extreme events.^4^

The choice of the process is, of course, very important and, in principle, should be empirically based. The way uncertainty enters into the model has implications for the solution of the underlying investment problem. Under certain circumstances, it is possible to have a closed form solution, as in the case of the Black and Scholes (BS, hereafter) formula (see e.g., Black and Scholes 1973, p. 644). Under other circumstances, it is necessary to resort to numerical methods such as binomial tree, trinomial and multinomial models, as well as Monte-Carlo and Least Squares Monte-Carlo simulations. When using a binomial tree, time is split into several intervals and the exercise of the option is evaluated at each step comparing the value of the exercise and the continuation value (Michailidis and Mattas 2007). This approach is less suitable when there are several sources of uncertainty (Regan et al. 2015; Schiel et al. 2018) and one may then resort to Monte-Carlo simulations (Boyle 1977). However, the use of Monte-Carlo simulations may be problematic when evaluating options that can be exercised at any time (Schiel et al. 2018). Longstaff and Schwartz (2001) proposed that Least Squares Monte-Carlo simulations, where the least squares method is used in order to estimate the expected continuation value at each time step, are used in such cases.

## Real-options analysis of adaptation and mitigation decisions

**Fig. 1:**
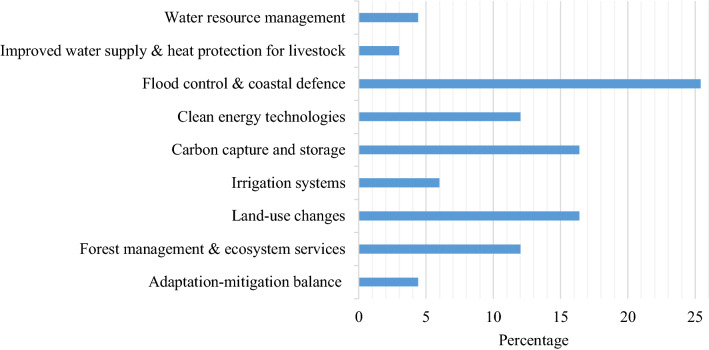
Distribution of studies with respect to the type of investment projects

The majority of the studies were published after 2009 and are undertaken on climate change mitigation. All the studies in our review are undertaken in developed countries. The majority (54%) of the papers focused on mitigation strategies (45% dealt with adaptation).^5^ In Fig. 1, the papers are categorized based on the type of mitigation or adaptation measure considered. We noticed that more than 25% of the studies, irrespective of whether focusing on mitigation or adaptation, considered investments in flood risk control and coastal defence. Other areas relatively frequently considered were investments in carbon capture and storage (CCS, hereafter) in energy production, and land-use change from cultivation of food and fiber crops to energy crops and agro-forestry (both above 16%). Concerning the agricultural sector, it is worth noting that only 6% of the studies dealt with adaptation strategies and that only investments in more efficient irrigation systems have been considered.

**Fig. 2:**
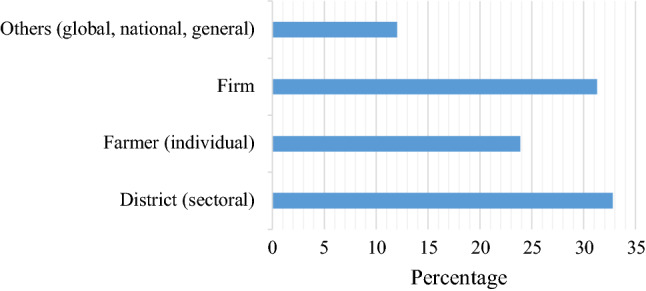
Unit of empirical analysis by the reviewed studies

To examine how risk preferences and strategic interactions have been incorporated in the reviewed papers, we looked first at the unit of analysis and then at the assumed objective of the agent. As indicated in Fig. 2, most of the studies (about 33%) dealt with decisions at district level or sectoral analysis. Decision-making by individual firms and households constituted 31% and 24%, respectively (see Fig. 2).

**Fig. 3:**
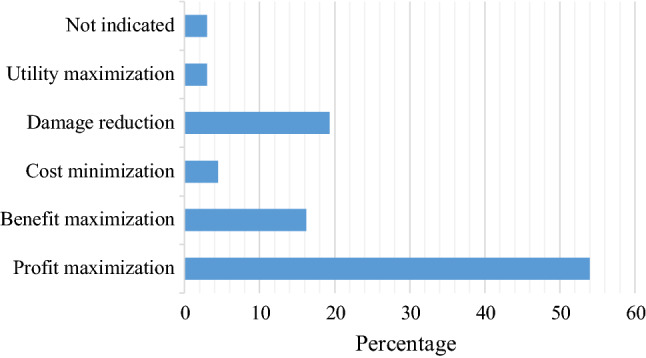
Underlying assumption for the objective of decision maker

As shown in Fig. 3, the majority (54%) of the reviewed studies that focus on decisions by individual firms or individual households assumed profit-maximizing behavior. Only three papers explicitly considered risk-aversion in climate adaptation and mitigation investment decisions. Out of these, two papers (3% of the studies), namely Narita and Quaas (2014) and Mense (2018), presented utility maximization approach to study farmers’ investment in an irrigation technology and residential relocation, respectively. Ihli et al. (2014) considered and measured farmers’ risk-preferences in an experimental study of investment in irrigation technology.

Only one of the reviewed papers considered strategic interaction. This unique exception is Narita and Quaas (2014) who explicitly considered the impact of coordination among farmers on their decision to invest in the exploitation of underground water for irrigation. They concluded that private decisions, in the absence of coordination about the common pool resource utilization, may lead to a socially sub-optimal choice of adaptation.

**Fig. 4:**
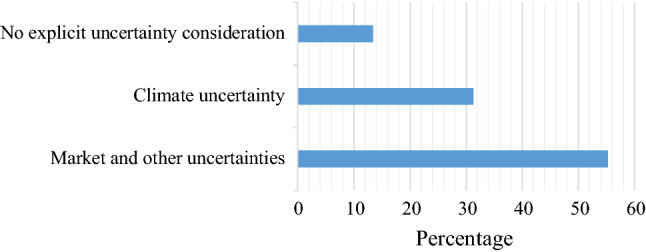
Types of uncertainty modeled in reviewed studies

The uncertainty characterizing climate change need to be taken into account when evaluating investments in climate actions. In our review, a majority of papers (more than 55%) considered uncertainty pertaining to markets and, in particular, to prices (see Fig. 4). As indicated in Fig. 4, only 31% of the reviewed papers explicitly included uncertainty related to climate change. In fact, more than 13% of the reviewed studies only discussed the possibility to apply real-options approach in the context of climate adaptation and mitigation investment analysis but did not include any empirical modelling of uncertainty.

How and to what extent climatic uncertainty and risk have been considered in the literature vary depending on the context. In the subsequent sections, we briefly present how different studies have modeled decisions to invest in climate adaptation and mitigation actions, and how these studies have taken into account climate uncertainty and risk preferences.

## Real-options analysis of investment in climate change adaptation

### Flood risk control and coastal defense

Several studies on flood risk control have considered different climatic scenarios to incorporate climate uncertainty.^6^ For example, Kind et al. (2018) formulated 500 scenarios for flooding in the case of a river discharge and assigned probabilities of each scenario occurring. These were then included in the objective function of the decision-maker deciding the optimal timing of an investment and the cost-minimizing size of a flood control facility. Based on the findings obtained using Monte-Carlo simulations and decision trees, the study suggested the need to design a flood control facility that has the capacity to handle high water discharges when fixed costs are high and alternatives for future adjustments are limited. Woodward et al. (2011) and Woodward et al. (2014) simulated flood risks and the infrastructure required to control flooding in the UK for the next 100 years based on the rise in sea level related to low, medium and high GHG emission scenarios. They used the equal probability for the three scenarios and concluded that incorporating flexibility into the decision for the investment in flood control facility allows taking uncertainty about future climate into account and increases the net benefits of the investment project. Ryu et al. (2018) calculated flood frequency under different climate scenarios in Global Circulation Models and assumed that flood damage linearly increases with the increase in flood frequency. Their results showed that adopting a real-options allowing for flexibility in planning improved the economic feasibility of the investment in flood control. Furthermore, Kim and Kim (2018) modelled the volatility of returns under the RCP4.5 and RCP8.5 climate scenarios of the IPCC’s representative concentration pathway. In the considered scenarios, they showed that investments in flood control facilities that would not have been undertaken based on a NPV criterion would be undertaken if option values were taken into account. In conclusion, these studies suggest that taking uncertainty and flexibility into account in investment decisions relating to flood control improves the effectiveness and efficiency of the infrastructure built to control flooding.

In addition to damages from the average expected events, it is important to account for climatic events with low probability of occurring but high impact when occured. Abadie et al. (2017) included climate uncertainty in the investment decision for flood risk control using a Poisson process capturing the impact of extreme climatic events. They combined Monte-Carlo simulations with two risk measures, Value at Risk (VAR) and Expected Shortfall (ES). The VAR and ES were used to quantify and incorporate the acceptable and unacceptable levels of risks in the investment decision, particularly when the variable of interest has a skewed distribution with high peak and heavy tail. Their results indicated that taking risk into account results in higher damage costs thus implying that it is beneficial to invest in flood control sooner than if risk is not taken into account.

Some of the studies on flood risk control focused on other types of uncertainty and did not explicitly consider climate uncertainty. Park et al. (2014), Kim et al. (2018) and Liu et al. (2018) emphasized the importance of market uncertainty and temporal flexibility when timing investments in flood control. They used binomial lattices, decision trees and Monte Carlo simulations as solution methods. An important insight from these studies is that the consideration of other options (such as the options to abandon, expand, contract, mothball, etc.), in addition to the option to invest, increased the value of the project considered. As indicated by Kim et al. (2018) this is because the higher operational flexibility implicitly provided by these options is valuable. This additional value may, as shown by Park et al. (2014), make it worth investing in projects that would not have been undertaken using the NPV criterion.

Studies focusing on investment in coastal defense infrastructure suggest that it is optimal to postpone investments. For example, Brown et al. (2018) simulated flood damages for the UK’s coastal nuclear power stations and found that investment in coastal defense should be postponed until 2090. They assumed a low probability of extreme storms occurring in the near future and limited damages for nuclear power plants. As their study did not investigate how sensitive are the results to changes in the underlying assumptions, they suggested future studies consider the sensitivity of the model to the time-evolving hazard mapping. In addition, Kim et al. (2018) concluded that immediate investment may be optimal only if the sea level rise above 4.9 mm per year. On the other hand, the results from Linquiti and Vonortas (2012), Kontogianni et al. (2014) and Oh et al. (2018) showed that the optimal timing of investment between different locations and thus highlights the need for location-specific analysis and policies.

### Water resources management

Several studies have considered climatic uncertainty when examining investment in water resource management. Erfani et al. (2018) assigned different probabilities for some likely scenarios of future precipitation and temperature for the UK based on UKCP09 climate projections and used a decision tree as the solution approach. They found that allowing for flexibility in the timing of investment of water management infrastructure increased the NPV with 6%. Using the binomial lattice approach in the Integrated Adaptive Model framework, Kim et al. (2017) investigated adaptation in hydropower generation and modeled the volatility of returns of investments based on RCP4.5 and RCP8.5 scenarios for future climate. They found that the optimal strategy was to postpone investment and that adaptation may significantly increase the annual generation of hydropower. Steinschneider and Brown (2012) studied investment in water management measures for adapting to climate change and used the forecasted hydro-climate variability to account for climatic uncertainty. Their results indicated that operational flexibility can serve as a robust adaptation mechanism in the management of water resources. Michailidis and Mattas (2007) considered investment in water resource management emphasizing the role of market uncertainty rather than that of climatic uncertainty. They used the binomial lattice method and found that the inclusion of temporal and operational flexibility can strongly affect the value associated with investments in irrigation dams.

### Agriculture and livestock adaptation to climate change

The only study analyzing investment for climate adaptation in agriculture that have incorporated climatic uncertainty in a real-options framework is Heumesser et al. (2012).^7^ They used a bio-physical process simulation model to investigate the investment in irrigation under the uncertain future precipitation. They assigned probabilities to 300 different possible levels of annual precipitation to reflect climatic uncertainty and computed the corresponding requirement of irrigation water. Their empirical analysis indicated that, even with high water prices, investment in water-saving irrigation would not be undertaken unless subsidies are paid.

A few studies have incorporated farmers’ risk preferences in decisions to invest in climate change adaptation in agriculture. For instance, Narita and Quaas (2014) studied the switch from rainfed to irrigated farming under uncertain crop yield (due to climate change) and captured farmers’ risk attitudes by using a relative risk aversion parameter in the Constant Relative Risk Aversion (CRRA) utility function. The study showed that climatic uncertainty may delay the decision to invest in irrigation by more than 40 years and that, without coordination, farmers with high risk aversion adapt too late, whereas farmers with low risk aversion adapt too early. Ihli et al. (2014) used economic experiment to examine investment in irrigation technology and incorporated farmers’ risk attitudes, elicited using a lottery game, in their model. They concluded that the real-options theory explains the decisions made by farmers better than the standard NPV analysis. They also found that postponing investment is optimal and that farmers learn over time from repeated decisions.

Other studies have focused on market uncertainty in modelling farmers’ investment decisions to adapt to climate change. For example, Schatzki (2003) examined how the volatility of the relative returns affected the decision of farmers to switch from crop production to forestry. Similarly, Sanderson et al. (2016) studied the switch from agriculture to cattle breeding focusing on the effect climatic conditions have on the returns from agriculture and cattle breeding. These studies indicated that higher volatility in relative returns reduces the likelihood of conversion.

## Real-options analysis in climate change mitigation

Studies on climate change mitigation seldom consider the effect of climatic uncertainty on investment decisions. Chesney et al. (2017) incorporated climatic uncertainty into mitigation investment decisions at the global level. They modelled global temperature dynamics using a GBM process and considered scenarios for baseline, moderate and severe warming or drying climates to simulate the green investment in biomass and wheat production. The results of their Monte Carlo simulations, under the assumptions of 1.5% discount rate, 0.3 °C temperature volatility and 14.75 °C global surface temperature, suggest that governments should invest 2.5% of GDP in mitigation.

### Investment in carbon capture and storage technologies

The studies on investments in CCS do not consider climatic uncertainty. Rather, they use different stochastic processes for prices and reach different conclusions about how market uncertainty affects the timing of investments. For instance, Zhu and Fan (2011) considered investment in CCS technologies with uncertain carbon and electricity prices and costs associated with the adoption of clean energy technologies. Zhu and Fan (2011) considered the variability in CCS technology costs as a measure of technological uncertainty, whereas Zhu and Fan (2013) assumed that electricity price follows an MR process while carbon price and technology operating costs evolve randomly following a GBM. They concluded that higher carbon prices and subsidies would foster the adoption of CCS technologies. Similarly, Fuss et al. (2008) and Chen et al. (2016) assumed that the price of carbon credits follows a GBM while electricity price follows an MR process. Fuss et al. (2008) found that higher volatility in electricity prices induce earlier adoption of CCS technology, whereas uncertainty surrounding government policy delays investment as it increases the volatility of carbon prices. Chen et al. (2016) concluded that subsidies may speed up the adoption of CCS technologies.

In general, the studies on the impact of carbon price and government policy uncertainties on the timing of investment in CCS provided conflicting results. In both Bose et al. (2013) and Kettunen et al. (2011), immediate investment in CCS technologies was optimal if the uncertainty in carbon price was low, while in Heydari et al. (2012) and Elias et al. (2018) immediate investment was optimal only in the presence of high carbon prices. In addition, Heydari et al. (2012) reported that the returns from CSS technologies were sensitive to variations in carbon prices. Finally, Hauck and Hof (2017) indicated that a higher carbon price and an extension in the regulatory deadline for the transition may significantly increase the investment in CCS technologies.

### Investments in clean technologies and renewable energy

None of the reviewed studies examining investment in clean and renewable energy considered climate uncertainty. They rather focused on market and policy uncertainty. Pless et al. (2016) evaluated investment in renewable energy using the BS formula under the assumption that natural gas price volatility follows an MR process. Using the BS formula and Monte-Carlo simulations, Sisodia et al. (2016) considered investment scenarios with different prices, subsidies and taxes. Shahnazari et al. (2017) and Schiel et al. (2018) evaluated the option to invest using Monte-Carlo simulations under the assumption that both market and policy uncertainty follow a GBM process. All of the above studies found that the option value increased with the volatility of market prices and government policy.

Some studies also emphasized the role of price and policy uncertainty when contemplating investments in cleaner technologies for energy production. Fuss et al. (2012) used dynamic programming to model investment in wind and biomass energy and solved the problem by resorting to Monte-Carlo simulations. They showed that lower prices of renewables discourage the adoption of cleaner technologies. Shahnazari et al. (2014) used Least Square Monte-Carlo simulations with a mean adjusting and reverting process to analyze the effect of volatile electricity prices and uncertainty related to the policy on investment in cleaner technologies for energy production. They considered the conversion of a coal plant into a clean production system and showed the negative effect of policy uncertainty on the decision to switch to cleaner technologies. Finally, Jang et al. (2013) evaluated investment in R&D of renewable energy using a binomial probability model and showed that considering the option value increased the return on the investment.

### Land-use change

Only one of the studies examining land-use change considered climatic uncertainty in modeling the investment decisions. Regan et al. (2017) considered wheat yields under three different climatic scenarios (baseline, moderate and severe warming or drying) in a study of farmers’ decision to switch from agriculture to biomass for energy production. They assumed that biomass, wheat prices and yield follow a GBM process and concluded that the conversion from traditional agriculture to biomass production is only economically profitable if the price of biomass is sufficiently high.

Several studies on land-use change have focused on market uncertainty. For example, Regan et al. (2015) looked at the same problem as Regan et al. (2017) but without considering climatic uncertainty. They assumed that gross margins for biomass production follow an ABM process and reported that switching from agriculture to energy feedstock production is profitable at the considered current market price. Regan et al. (2015) found that the rates of return triggering the switch are higher than in a standard NPV analysis. Similarly, Behan et al. (2006) examined the decision by farmers to switch from agriculture to forestry using dynamic programming. Their results indicate that, given the current conditions, it would be optimal to wait approximately six years before switching. Di Corato et al. (2013) studied the conversion of agricultural land to energy forestry in Sweden. They assumed that profits from agriculture evolve randomly following a GBM process and concluded that immediate investment is conditional on having a subsidy covering of at least 75% of the cost of establishing the plantation. In the context of Amazonian deforestation, Di Corato et al. (2018) assumed that the economic benefits from forest conservation follow a GBM process and studied the timing of conversion of forestland to agricultural land. They showed that higher and less volatile forest benefits may deter deforestation. This indicates that economic incentives for forest conservation must not only be higher but also more stable over time.

Another group of studies also emphasized market uncertainty in studying farmers’ decisions to switch from agriculture to forestry. Frey et al. (2013) assumed that crop returns, timber and pecan prices followed an MR process and used aggregate time-series data. Their results from the Monte-Carlo simulations indicate that forestry and agroforestry are less profitable than agriculture. Similarly, Hauer et al. (2017) assumed that ethanol prices follow an MR process while land prices follow a GBM process. Using Least Square Monte-Carlo simulations, they showed that a significant subsidy is required in order to induce a switch from agriculture to energy forestry. In both Song et al. (2011) and Yemshanov et al. (2015), the underlying conversion problem was solved using the collocation and lattice simulation method. In Song et al. (2011), a return from the energy crop followed an MR and a GBM process and they concluded that it is optimal for farmers to postpone the conversion of land from soybean to energy crop production. In Yemshanov et al. (2015), land values follow a GBM process. They showed that, when comparing the amount of land to be considered for afforestation, taking uncertainty into account results in an afforested area smaller than if the standard NPV criterion is applied.

### Sustainable forest management and ecosystem services

The studies on forest management and ecosystem services typically do not consider climate uncertainty. One exception is Schou et al. (2015) who incorporated climate uncertainty in the modelling of decision to invest in forestry and forest regeneration. They considered the distribution of climate impacts across the scenarios and the subjective probability of the forest managers about the possible climate scenarios (assuming that decision-makers update their beliefs by using the Bayes’ rule). Their results indicate that forest managers make sub-optimal harvest decisions if they believe that climate change uncertainty will prevail for a longer period.

Only Mense (2018) incorporated the effects of risk-aversion in the investment decision in the context of ecosystem services. He used utility maximization approach for the payments to ecosystem services in a case study of relocation to less polluted areas. The results suggest that the value of amenity increases with risk aversion and uncertainty concerning environmental quality.

The remaining studies in this category do not consider climate uncertainty in their modelling. For example, Chladná (2007) studied the same problem as Schou et al. (2015) but considered only market uncertainty by assuming that wood prices follow an MR process and that carbon prices follow a GBM process. The optimal rotation period was found to decrease with the increase in discount rate and the responsibility of forest owners with respect to the reduction of CO2 released when harvesting. Last, Matsuhashi et al. (2008) investigated the impact of the Clean Development Mechanism (CDM) using dynamic programming. They modeled the evolution of the price of certified emission reductions (CER) resulting from a CDM project as a GBM process and showed how a fixed price for CER may allow hedging against fluctuating revenues. Srinivasan (2015) used the bounded random walk methodology and suggested that there is an option value of 3.8–6.5% for investments in ecosystem conservation in India.

Insley (2002) allowed for timber prices to follow either an MR or a GBM process and solved the problem using the implicit finite difference approach. She found that the option value increased due to price volatility and that assuming a MR process rather than a GBM has a significant impact on the optimal tree cutting policy. Using the binomial tree approach and modelling the evolution of carbon and timber prices with MR processes, Tee et al. (2014) found that the yield under carbon forestry was 73% higher than under commercial forestry. Milanesi et al. (2014) proposed a Fuzzy payoff model for the analysis of forest establishment investment and showed that some positive option value would be ignored if the investment was evaluated using the traditional NPV approach. Sauter et al. (2016) conducted an economic experiment testing whether harvesting decisions reflected the predictions of the Faustmann rule rather than the real-options approach. They found that farmers seem relatively more inclined towards following a timing rule accounting for the presence of an option value.

## Discussion and conclusions

This paper systematically reviews the literature using a real-options approach for the evaluation of investment in climate change adaptation and mitigation. It indicates that real-options analysis is a useful tool for investment appraisal under uncertainty. This is because the analysis takes the timing of investments and operational flexibility into account. The paper also reveals gaps in the literature and identifies areas for future research. The review shows that the majority of papers focus on climate mitigation rather than climate adaptation. This result seems to be in line with the IPCC (2014d) reporting that less emphasis has been given to adaptation. The important role climate adaptation can play in reducing the impact of climate change is now widely recognized and more studies applying a real-options framework to investments in adaptation strategies could be expected in the future.

In terms of mitigation strategies, investment in clean energy technologies and land-use changes, particularly the switch to bioenergy production, have been widely investigated. This is, once again, in line with the emphasis, indicated by IPCC (2014d, e), on investments in low-carbon and carbon-neutral energy technologies, not only to reduce GHG emissions but also to lower the long-term costs of mitigation. Other measures, such as alternative farming practices and cropland management, a reduction in the use of chemical fertilizers, and the restoration of organic soils, can also be of interest to farmers aiming at adapting to or mitigating climate change (IPCC 2014a, c). However, this review indicates that no studies examining these measures has yet been published. We have also noticed that there is no application of real-options analysis to investment in climate adaptation and mitigation in the agriculture sector of developing countries. This is quite peculiar considering that the negative impacts of climate change is exacerbated in developing countries (IPCC 2014d, e).

Climate-driven uncertainty, affecting temperature, rainfall and yields, needs further investigation, as it has barely been considered in previous studies. The majority of the studies considered focus on market and policy uncertainty when examining investment in climate adaptation and mitigation strategies. Specifically, market input and output prices and carbon price volatilities are usually considered. However, the proper consideration of climatic uncertainty may be crucial to inform policy interventions concerning investments in measures to cope with climate change (Quiggin 2008; Heal and Millner 2014; IPCC 2014e).

Moreover, previous studies have assumed that decision-makers are risk-neutral and that they maximize profits. Profit maximization is standard when modelling firms’ behavior in the economic literature. However, not taking risk preferences into account in real-options models can lead to over or underestimation of the magnitude of investments (Isik 2005). Chronopoulos et al. (2011) showed that agents being risk averse may further delay investment. Furthermore, focusing on the farming sector, profit maximization is not necessarily consistent with farm production choices at household level. This may be the case, for instance, when considering the existence of duality between consumption and production, market incompleteness and risk avoiding behaviors (Mendola 2007; LaFave and Thomas 2016). In this regard, considering risk-aversion can improve the understanding of individual actions pertaining to climate change adaptation and mitigation.

Furthermore, the reviewed studies have overlooked the impact of strategic interaction. Decision-makers are generally assumed to be independent. In reality, however, firms make decisions considering competition from other firms. Similarly, farmers’ decisions can be affected by the actions and decisions of their neighbors, and vice versa. Moreover, climate change is, in several respects, a problem of collective action, where the independent actions of an individual may not necessarily be effective (IPCC 2014d). This means that it may be important to take decision-makers' strategic interactions into account when modelling investments in adaptation and mitigation. It can better inform policies pertaining to climate change adaptation and mitigation.

To summarize, this paper highlights some key implications for future research. One interesting area for future research is to incorporate uncertainties pertaining to climate change into the real-options analysis of investments in adaptation and mitigation actions. It is also important to apply the real-options approach to investigate the feasibility of climate-friendly measures in the agricultural sector in the context of low-income countries. Moreover, it is also crucial to consider the duality and risk-averse behavior of farm households in the real-options analysis. This can capture the unique nature of farm households as consumers and producers. Incorporating the effects of strategic interaction in resource use in rural settings into the real-options analysis is also an interesting area for future studies concerning the formulation of policies coping with climate change.

## Electronic supplementary material

Below is the link to the electronic supplementary material.

Supplementary material 1 (PDF 1202 kb)

## Footnotes

1 See Dixit and Pindyck (1994) for an illustration of the theory of optimal investment under uncertainty and irreversibility.

2 The string ‘climat*’ also includes ‘climate’ and ‘climatic’, whereas ‘resilien*’ also includes ‘resilient’ and ‘resilience’.

3 See Appendix S2.

4 See Dixit and Pindyck (1994, Ch. 3) for an illustration of the properties of these stochastic processes.

5 Only one paper, i.e. Maybee et al. (2012), deals with the adaptation-mitigation balance (see Appendix S1 in the supplementary material).

6 See Gersonius et al. (2015) and Manocha and Babovic (2018) for a discussion of the suitability of the real-options approach for the analysis of investments in flood risk control infrastructure.

7 See Hertzler (2007), Dobes (2008) and Dittrich et al. (2017) for a discussion of the suitability of the real-options approach for the analysis of investments in climate adaptation in the agricultural sector.
