# Correction to: Investing in climate change adaptation and mitigation: A methodological review of real-options studies

**DOI:** 10.1007/s13280-021-01700-0

**Published:** 2022-01-29

**Authors:** Tsegaye Ginbo, Luca Di Corato, Ruben Hoffmann

**Affiliations:** 1 Department of Economics, Swedish University of Agricultural Sciences, Box 7013, 75007 Uppsala, Sweden; 2 Department of Economics, Ca’ Foscari University of Venice, Cannaregio 873, Fondamenta San Giobbe, 30121 Venice, Italy

## Correction to: Ambio (2021) 50:229–241 10.1007/s13280-020-01342-8

The third cell in Row 42 in Appendix S3 (currently Appendix S2) of the Supplementary Material erroneously skipped blank, and the subsequent rows 43–67 (pages 10–11) swapped during the revision process. The correct version (Appendix S2) of the Supplementary Material is provided in this correction.

The original article has been corrected.

## Supplementary Information

Below is the link to the electronic supplementary material.

Supplementary file1 (PDF 1202 kb)

