# Supplementary material for: Investing in climate change adaptation and mitigation: A methodological review of real-options studies
Source: Ambio. 2020 May 25;50(1):229–41. doi: 10.1007/s13280-020-01342-8 (PMC7708567; doi:10.1007/s13280-020-01342-8)
Supplement: Supplementary file 1 — Supplementary material 1 (PDF 1202 kb) [file 13280_2020_1342_MOESM1_ESM.pdf]

Ambio

Electronic Supplementary Material

This supplementary material has not been peer reviewed

Title: **Investing in climate change adaptation and mitigation - A methodological review of real-options studies**

Tsegaye Ginbo, Luca Di Corato, Ruben Hoffmann

Appendix S1. Description of the studies

Although 1973 was used as starting year for our literature survey, all of the papers finally selected were published after 2001 and the overwhelming majority of the studies (86 percent) were published after 2009 (see Fig. S1). This indicate that the real-options approach started to be adopted in analyses of investments in climate change adaptation and mitigation relatively recently.

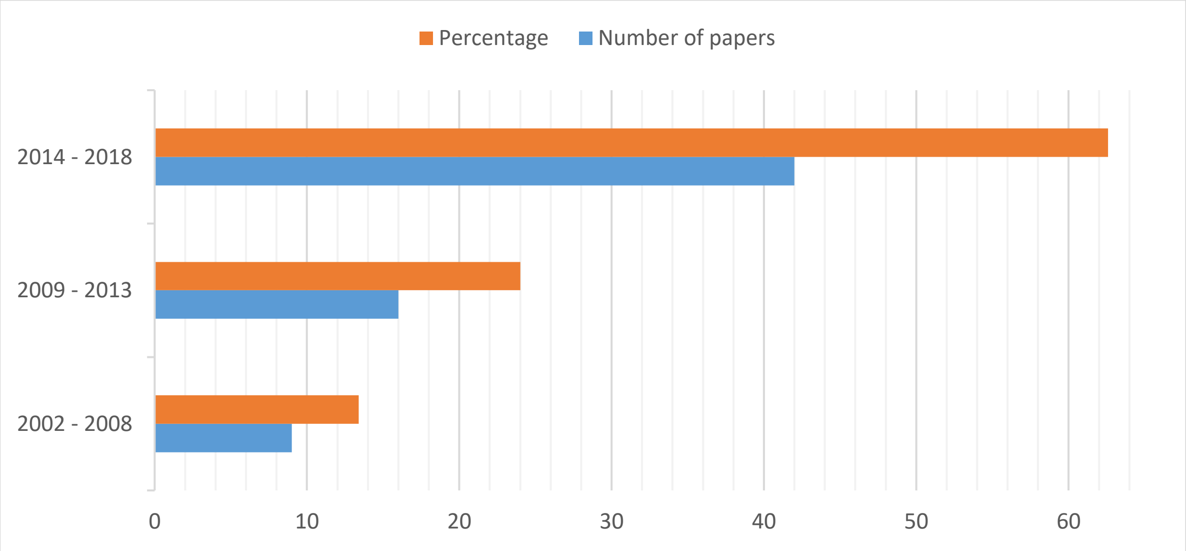

Fig. S1: Number of studies across different periods

Another issue worth considering is the composition of studies in terms of adaptation and mitigation. This can also provide an important insight into the subject of focus for previous studies. As depicted in Fig. S2, the majority of the studies dealt with climate change mitigation actions. Specifically, 54 percent of selected studies have exclusively applied real options analysis in climate change mitigation whereas only 45 percent of papers address adaptation actions (see Fig. S2). The remaining 1.5 percent (only one) study dealt with the comparison and combination of adaptation and mitigation policies.

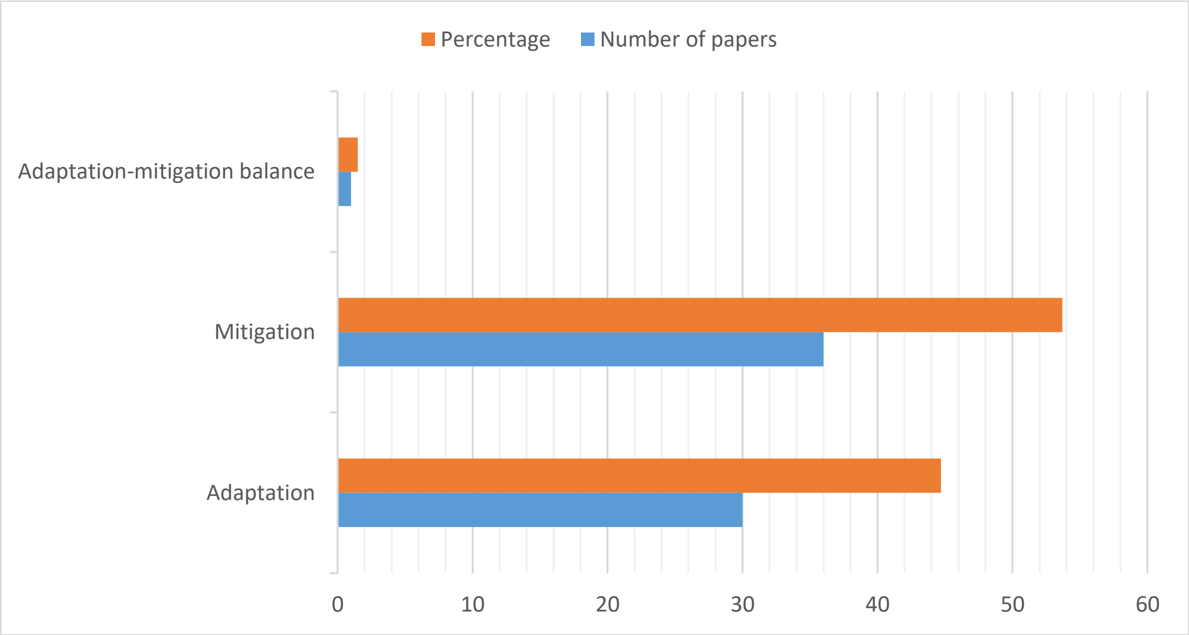

Fig. S2: Percentage of publications in adaptation and mitigation

In terms of the regional coverage of the studies, our review reveals the dominance of the few developed countries in the distribution of studies. UK (over 13 percent), Australia (12 percent), South Korea (over 10 percent) are top three countries where most of the reviewed studies conducted, followed by Germany with 9 percent (see Fig. S3).

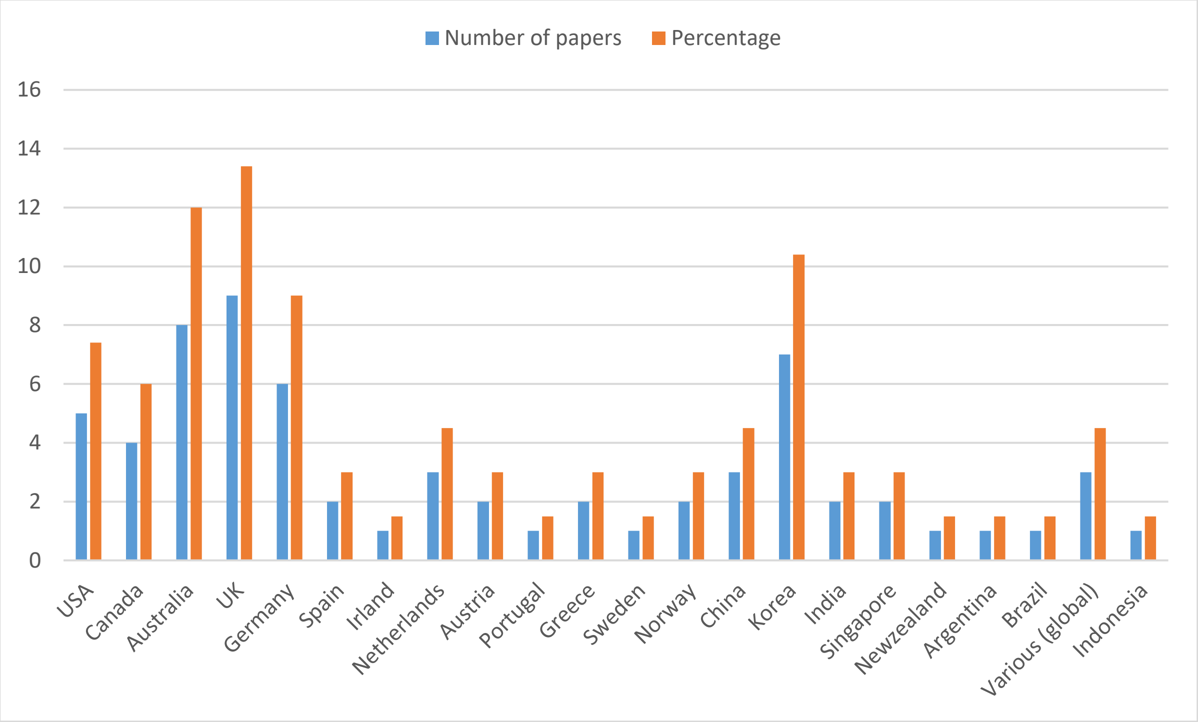

Fig. S3: Distribution of studies across countries

On the other hand, studies in this review are interdisciplinary in the sense that they cover a wide-range of research areas. Specifically, the papers are published in 42 academic journals where Journal of Cleaner Production is main journal which publish 5 articles followed by Applied Energy, Forest Policy and Economics, and Journal of Cleaner Production journals each publishing 4 articles. However, most of the journals (29 out of 42) publish only one article. In terms of the study subjects, energy and forest economics are among the main research areas covered by the reviewed papers.

Our review reveals that Monte-Carlo simulations, dynamic programming and binomial lattice are the most commonly used solution methods. As illustrated in Fig. S4, roughly 24 percent of the reviewed papers used Monte-Carlo simulations, 18 percent used dynamic programming and, 17 percent used lattice methods. About 10 percent of the papers simply discussed the applicability and suitability of a real-options analysis relative to other methods for the valuation of investments.

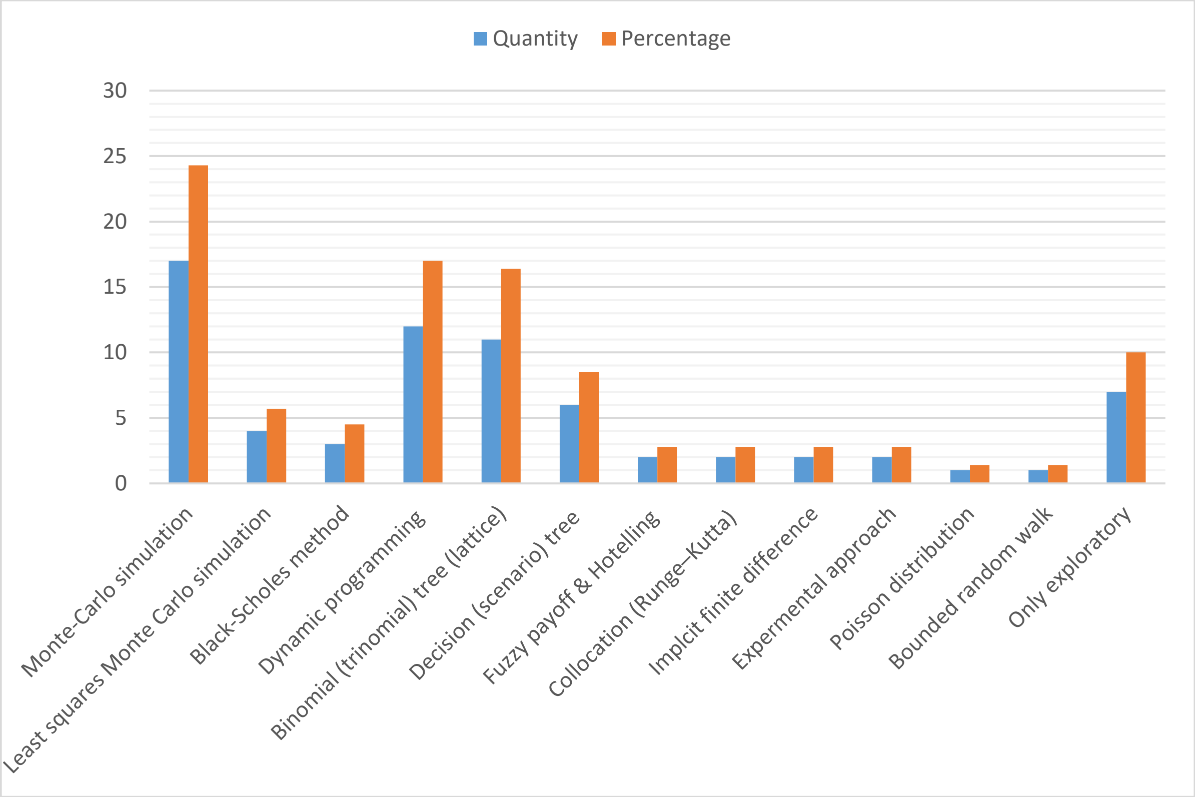

Fig. S4: Solution methods used in reviewed studies

In some studies, a combination of methods has been adopted in order to increase the robustness of the results obtained. For example, Woodward et al. (2014) and Kind et al. (2018) used Monte-Carlo simulations and decision trees, Chen et al. (2016) used Monte-Carlo simulations and dynamic programming while Sisodia et al. (2016) used BS and Monte-Carlo simulations, and Shahnazari et al. (2017) used Monte-Carlo simulations and portfolio optimization methods. Other studies have used econometric methods to test the theoretical predictions of real-options models. For example, Schatzki (2003) used limited dependent variable analysis, Behan et al. (2006) used panel data regression approaches while Heumesser et al. (2012) and Park et al. (2014) used linear regression methods. Finally, in order to overcome the lack of empirical data, Ihli et al. (2014) and Sauter et al. (2016) generated the required data through experiments.

References cited in Appendix S1

Behan, J., K. McQuinn, and M. J. Roche. 2006. Rural land use: Traditional agriculture or forestry? Land Economics **82**:112-123.

Chen, H., C. Wang, and M. Ye. 2016. An uncertainty analysis of subsidy for carbon capture and storage (CCS) retrofitting investment in China's coal power plants using a real-options approach. Journal of Cleaner Production **137**:200-212.

Heumesser, C., S. Fuss, J. Szolgayová, F. Strauss, and E. Schmid. 2012. Investment in Irrigation Systems under Precipitation Uncertainty. Water Resources Management **26**:3113-3137.

Ihli, H. J., S. C. Maart-Noelck, and O. Musshoff. 2014. Does timing matter? A real options experiment to farmers' investment and disinvestment behaviours. Australian Journal of Agricultural and Resource Economics **58**:430-452.

Kind, J. M., J. H. Baayen, and W. J. W. Botzen. 2018. Benefits and Limitations of Real Options Analysis for the Practice of River Flood Risk Management. Water Resources Research **54**:3018-3036.

Park, T., C. Kim, and H. Kim. 2014. Valuation of Drainage Infrastructure Improvement Under Climate Change Using Real Options. Water Resources Management **28**:445-457.

Sauter, P. A., O. Mußhoff, B. Möhring, and S. Wilhelm. 2016. Faustmann vs. real options theory - An experimental investigation of foresters' harvesting decisions. Journal of Forest Economics **24**:1-20.

Shahnazari, M., A. McHugh, B. Maybee, and J. Whale. 2017. Overlapping carbon pricing and renewable support schemes under political uncertainty: Global lessons from an Australian case study. Applied Energy **200**:237-248.

Sisodia, G. S., I. Soares, and P. Ferreira. 2016. Modeling business risk: The effect of regulatory revision on renewable energy investment - The Iberian case. Renewable Energy **95**:303-313.

Woodward, M., Z. Kapelan, and B. Gouldby. 2014. Adaptive flood risk management under climate change uncertainty using real options and optimization. Risk Analysis **34**:75-92.

Appendix S2: Extracted information from the papers selected for the analysis

I. Focus, sector, methods, unit of analysis and underlying assumptions

s.n	Paper	Focus	Sector	Method (s)	Unit of analysis	Underlying assumption (s)
1.	Abadie et al 2017	adaptation	urban flood risk management	Monte Carlo simulation	District	Damage minimization
2.	Behan et al 2006	mitigation	agr-forestry switching	Dynamic programming, Panel data econometrics	Farmer	Profit mazimization
3.	Bose et al 2013	mitigation	Carbon capture and storage	Black-Scholes method	Firm	Profit mazimization
4.	Brown et al 2018	adaptation	Coastal defence	Monte Carlo simulation	District	Damage minimization
5.	Buurman and Babovic 2016	adaptation	adaptation policy	Exploratory	National	Damage minimization/benefit maximization
6.	Chen et al 2016	mitigation	Carbon capture and storage in power generation	Monte Carlo simulation, Backward stochastic dynamic programming	Firms /sector	Profit mazimization
7.	Chesney et al 2016	mitigation	global	Monte Carlo simulation	Global	Benefit maximization (global GDP)
8.	Chladná 2007	mitigation	forestry	Dynamic programming	Forest owner	Profit mazimization
9.	Di Corato et al 2013	Mitigation	agri-energy forestry switching	Dynamic programming	Farmer	Profit mazimization
10.	Di Corato et al 2018	mitigation	forest conversion	Dynamic programming	Social planner	Maximize social benefit (welfare)
11.	Dittrich et al 2017	adaptation	livestock	Exploratory	General	Benefit maximization
12.	Dobes 2008	adaptation	various sectors	Exploratory	General	Benefit maximization
13.	Elias et al 2018	mitigation	Carbon capture and storage in power generation	Bivariate lattice	Firm	Profit mazimization

Appedix S2: Continued

S.no.	Paper	Focus	Sector	Method (s)	Unit of analysis	Underlying assumption (s)
14.	Erfani et al 2018	adaptation	water resources management	scenario tree, AND	District	Cost minimization
15.	Frey et al 2013	mitigation	agri to forestry-agroforestry switching	Monte Carlo simulation	Farmer	Reward maximization
16.	Fuss et al 2012	mitigation	renewable energy	Dynamic programming	Firm	Cost minimization
17.	Fuss et al 2008	mitigation	Carbon capture technologies, in electricity sector	Monte Carlo simulation	Firm	Profit mazimization
18.	Gersonius et al 2015	adaptation	Flood risk managemnt	Exploratory	General	damage minimization
19.	Hauck and Hof 2017	mitigation	Carbon capture and storage	discrete time approximation, of GBM	Firm	Profit mazimization
20.	Hauer et al 2017	mitigation	agri to energy forestery	Least squares Monte Carlo simulation	Farmer	Profit mazimization
21.	Hertzler 2007	adaptation	various sectors	Exploratory	Farmers	damage minimization
22.	Heumesser et al 2012	adaptation	irrigation	Dynamic programming	Farmer	Profit mazimization
23.	Heydari et al 2012	mitigation	Carbon capture and storage (Energy)	Dynamic programming	Firm	Profit mazimization
24.	Ihli et al 2013	adaptation	irrigation	Expermental	Farmer	Profit mazimization
25.	Insley 2002	mitigation	Forestry	Implicit finite difference	Forest owner	Profit mazimization
26.	Jang et al 2013	mitigation	renewable energy technologies	binomial probability model	Firm	Profit mazimization
27.	Kettunen et al 2011	mitigation	CSS	Binomial scenario tree	Firm	Profit mazimization
28.	Kim et al 2017	adaptation	urban infrastructure (erosion contrl)	binomial lattice approach	District	Damage minimization
29.	Kim et al 2017	adaptation	energy (hydropower)	binomial lattice	District	Damage minimization
30.	Kim and Kim 2018	adaptation	coastal defence	binomial lattice	Firm	Profit mazimization
31.	Kim et al 2018	adaptation	flood control facility	Simulations	District	Damage minimization

Appendix S2: Continued

s.n	Paper	Focus	Sector	Method (s)	Unit of analysis	Underlying assumption (s)
32.	Kind et al 2018	adaptation	coastal defence	Monte Carlo simulation, decision tree	District	Not indicated
33.	Kontogianni et al 2014	adaptation	coastal defence	Monte Carlo simulations	District	Damage minimization
34.	Linquiti and Vonortas 2012	adaptation	river flood risk management	Monte Carlo simulation	District	Damage minimization
35.	Liu et al 2017	adaptation	urban flood risk management	trinomial tree model (extension of lattice binomial model)	District	Not indicated
36.	Manocha and Babovic 2018	adaptation	infrastructure	Decision tree	District	minimize damage/maximize benefits
37.	Manocha and Babovic 2018	adapation	storm water management	Dynamic programming	District	minimize damage/maximize benefits
38.	Marques et al 2015	mitigation	water distribution system	Hotelling methodology	District	Cost minimization/GHG minimization
39.	Matsuhashi et al 2008	mitigation	CDM	Exploratory	Firm (investor)	Profit mazimization
40.	Maybee et al 2012	mitigation	Payment for Ecosystem Services	Exploratory	General	Profit mazimization
41.	Mense 2017	Mitigation	Payment for Environemntal quality	Dynamic programming	Individual resident	Utillity maximization

Appendix S2: Continued

s.n	Paper	Focus	Sector	Method (s)	Unit of analysis	Underlying assumption (s)
42.	Michailidis and Mattas 2007	adaptation	Water resourses management	Binomial lattice method	National	Profit mazimization
43.	Milanesi et al 2014	mitigation	forest investement	Fuzzy pay off model	District	Profit mazimization
44.	Narita and Quaas 2014	adaptation	Irrigation	Dynamic programming	Farmers	Profit mazimization
45.	Oh et al 2018	adaptation	coastal defence	Rainbow option method (Quadrinomial lattice)	District	Damage minimization
46.	Park et al 2014	adaptation	Drainage infrastructure	binomial model	District	Benefit maximization
47.	Pless et al 2016	adaptation	agriculture	Black-Scholes method	Firm	Profit mazimization
48.	Regan et al 2017	mitigation	agriculture - to biomas	Monte Carlo simulation	Farmer	Profit mazimization
49.	Regan et al 2015	mitigation	renewable vs natural gas energy	Monte Carlo simulation	Farmer	Profit mazimization
50.	Ryu et al 2018	adaptation	flood risk management	binomial tree model	District	Benefit maximization
51.	Sanderson et al 2015	adaptation	agriculture (wheat to sheep switching)	Dynamic programming	Farmer	Profit mazimization
52.	Sauter et al 2016	mitigation	forestry	Expermental	Forest owner	Profit mazimization
53.	Schatzki 2003	mitigation	agriculture - bio-energy feedstock	Runge–Kutta method, econometric analysis	Farmer	Profit mazimization

Appendix S2: Continued

S.N	Paper	Focus	Sector	Method (s)	Unit of analysis	Underlying assumption (s)
54.	Schiel et al 2018	mitigation	emission abatement	Monte Carlo simulation	Firm	Profit mazimization
55.	Schou et al 2015	mitigation	Forest regeneration	Dynamic programming	Farmer	Profit mazimization
56.	Shahnazari et al 2014	adaptation	agriculture to forest switching	Least squares Monte Carlo simulation	Firm	Profit mazimization
57.	Shahnazari et al 2017	mitigation	clean energy transition	Monte Carlo simulation, portfolio optimisation	Firm	Profit mazimization
58.	Sisodia et al 2016	mitigation	clean energy	Black-Scholes method, Monte-carlo simulation	Firm	Profit mazimization
59.	Srinivasan 2015	mitigation	Ecosystem conservation	Bounded random walk	Farmer	Benefit maximization
60.	Song et al 2011	mitigation	renewable energy	Collocation method	Farmer	Profit mazimization
61.	Steinschneider and Brown. 2012	adaptation	Water resources management	Simulations	District	Benefit maximization
62.	Tee et al 2014	mitigation	forestry	binomial tree	District	Profit mazimization
63.	Woodward et al 2011	adaptation	Flood risk management	Monte Carlo simulation	District	Benefit maximization
64.	Woodward et al 2014	adaptation	Flood risk management	Monte Carlo simulation, decision trees	District	Benefit maximization
65.	Yemshanov et al 2015	mitigation	agri-forestry switching	Lattice simulatios, with bioeconomic model	District	Profit mazimization
66.	Zhu and Fan 2011	mitigation	carbon capture and storage	Least squares Monte Carlo simulation	Firm	Profit mazimization
67.	Zhu and Fan 2013	mitigation	carbon capture and storage	Least squares Monte Carlo simulation	Firm	Profit mazimization

Appendix S2: Continued

II. Uncertainty type, stochastic processes and case studies

S.No	Paper	How they accounted for uncertainty	Uncertainty considered	Stochastic process (es)	Country	Journal
1.	Abadie et al 2017	risk measures (VAR, and ES) using MC simulation, and compare it with poission dist of prob of extreme events	Climate change	Geometric Brownian motion	Spain	Environmental Modelling & Software
2.	Behan et al 2006	Market uncertainity (prices) No treatment of cc uncertainity.	Market	NI	Irland	Land Economics
3.	Bose et al 2013	No treatment of cc uncertainity. They focused on uncertainity in macroeconomic variables	Market	NI	India	International journal of regulation and governance
4.	Buurman and Babovic 2016	Indicate that ROA deal with statistical, quantifiable uncertainty, but not deep uncertainity	Market	NI	South Korea	Policy and Society
5.	Chen et al 2016	Uncertainty in prices (carbon coal, and electricity prices)	Market	Geometric Brownian motion & MR	China	Journal of Cleaner Production
6.	Chesney et al 2016	Temperature process dynamics modeled as BM, in addition to global GDP process	Market	BM	global	Ann Oper Res
7.	Chladná 2007	Wood price modeled as MR , carbon prices as GBM	Market; climate policy	Geometric Brownian motion & MR	Austria	Forest Policy and Economics
8.	Di Corato et al 2013	Profit per hectare as GBM	Market	Geometric Brownian motion	Sweden	Forest Policy and Economics
9.	Di Corato et al 2018	Forest benefits as GBM	Market	Geometric Brownian motion	Brazil	Environment and Development Economics
10.	Dittrich et al 2017	No explicit treatment of uncertainity	No	NI	UK	Reg Environ Change
11.	Dobes 2008	No explicit treatment of uncertainity	No	NI	Australia	Agenda

Appendix S2: Continued

S.No	Paper	How they accounted for uncertainty	Uncertainty considered	Stochastic process (es)	Country	Journal
12.	Elias et al 2018	Natura gas and electricity prices as MR	Market	MR	Canada	Journal of Cleaner Production
13.	Erfani et al 2018	Scenario tree to appro distribution of water supply	Climate change	NI	UK	Water Resources Research
14.	Frey et al 2013	Estimate MR model of crop returns, and timber and pecan prices using aggregate time-series data	Market	MR	USA	Agricultural Economics
15.	Fuss et al 2012	Stochastic carbon price	Market; climate policy	NI	Germany	Energy Policy
16.	Fuss et al 2008	Electricity price as MR, and Carbon price as GBM *(market and climate policy uncertainties)	Market; climate policy	Geometric Brownian motion & MR	Germany	Applied Energy
17.	Gersonius et al 2015	No explicit treatment of uncertainity	No	NI	Netherlands	J Flood Risk Management
18.	Hauck and Hof 2017	Gas price and carbon price uncertanity as GBM	Market	Geometric Brownian motion	Netherlands	Energy Policy
19.	Hauer et al 2017	Ethanol price as MR, and agri land price as GBM	Market	Geometric Brownian motion	Canada	Canadian Journal of Agricultural Economics
20.	Hertzler 2007	No explicit treatment of uncertainity	No	NI	Australia	Australian Journal of Agricultural Research
21.	Heumesser et al 2012	Incroporated uncertainity in future precipitation in bio-physical proccess simulation model (EPIC)	Climate change	NI	Austria	Water Resour Manage
22.	Heydari et al 2012	Fuel input price, electricity output price and carbon permit price uncertainties as GBM	Market; climate policy	Geometric Brownian motion	UK	Computational Management Science

Appendix S2: Continued

S.No	Paper	How they accounted for uncertainty	Uncertainty considered	Stochastic process (es)	Country	Journal
23.	Ihli et al 2013	No explicit treatment (specification) of uncertainity	No	Arithmetic Brownian motion	Germany	Australian Journal of Agricultural and Resource Economics
24.	Insley 2002	Uncertainty of timber price as MR and GBM	Market	MR	Canada	Journal of Environmental Economics and Management
25.	Jang et al 2013	Price (market) uncertainity as MR and R&D uncertainty as Binomial prob. Model	Market	Geometric Brownian motion & MR	Korea	International Journal of Energy Research
26.	Kettunen et al 2011	Carbon price uncertainty	Market, climate policy	MR	UK	The energy journal
27.	Kim et al 2017	No explicit treatment of uncertainity	No	NI	South Korea	Journal of Cleaner Production
28.	Kim et al 2017	Volatility of project returns based on Future climate scenarios, up and downward mov\t of adaptation benefits (cash flow)	Climate change	Geometric Brownian motion	South Korea	Journal of Cleaner Production
29.	Kim and Kim 2018	Volatility of project returns based on Future climate scenarios, up and downward mov\t of adaptation benefits (cash flow) and risk neutral prob	Climate change	Geometric Brownian motion	South Korea	Sustainability
30.	Kind et al 2018	Discharge scenario in decsion tree (minimize expexted invesment cost over all scenarios)	Climate change	NI	Netherlan ds	Water Resources Research

Appendix S2: Continued

S.No	Paper	How they accounted for uncertainty	Uncertainty considered	Stochastic process(es)	Country	Journal
31.	Liu et al 2017	Uncertainity of rainfall Using possible changes in system for 3 jump parameters (up, down, same mov'ts) with related transition prob for trinomial model, leading to adapatation cost, drainage capacity and uncertainities	Climate change	NI	UK	Nat Hazards
32.	Manocha and Babovic 2018	No explicit treatment of uncertainity	No	NI	Portugal	Water
33.	Manocha and Babovic 2018	Certified emission reduction values as GBM	Market; climate policy	NI	Various, Asian	Environmental Science and Policy
34.	Marques et al 2015	No explicit treatment of uncertainity	No	NI	Australia	Journal of Hydroinformatics
35.	Matsuhashi et al 2008	Use a multi-objective optimization approach in adapation pathway to identify the set of preferred pathways (solutions) that are able to cater to deep uncertainty	Climate change	Geometric Brownian motion	Singapore	Environmental Economics and Policy Studies
36.	Maybee et al 2012	No explicit treatment of uncertainity	No	NI	Singapore	Economic Papers
37.	Michailidis and Mattas 2007	Project value as GBM, Binomial model with risk neutral probabilities p, 1-p for up and downward mov't	Market	Geometric Brownian motion	Greece	Water Resour Manage
38.	Milanesi et al 2014	Different scenarios for project value or income	Market	NI	Argentina	Fuzzy Economic Review
39.	Narita and Quaas 2014	Volatility (uncertainty) in agricultural productivity as GBM	Market	Geometric Brownian motion	Germany	Climate Change Economics

Appendix S2: Continued

S.No	Paper	How they accounted for uncertainty	Uncertainty considered	Stochastic process (es)	Country	Journal
40.	Park et al 2014	Volatiltiy in value of a drainage system (the uncertainty associated with investments in drainage infrastructure under climate change) is estimated from historical flood damages	Climate change	NI	Korea	Water Resour Manage
41.	Pless et al 2016	Market uncertainty (natural gas price as Geometric MR and price of renewable energy)	Market	Geometric Ornstein-Uhlenbeck	USA	Energy Policy
42.	Regan et al 2017	Use different climate scenario (baseline, moderate and severe warming or drying), calculate wheat yield for scenario, characterize biomas and wheat price volatility (GBM) together with variable yield	Climate change	Geometric Brownian motion & MR	Australia	Journal of Environmental Management
43.	Regan et al 2015	Price uncertainties ()	Market	Arithmetic Brownian motion	Australia	Journal of Environmental Management
44.	Ryu et al 2018	Dirrerent climate scenario (up, down and basecase) for binomial model, using Globla Circulation model (GCM) datasets, conduct flood frequency analys for all GCM	Climate change	NI	Korea	Mitig Adapt Strateg Glob Change
45.	Sanderson et al 2015	Returns as MR	Market	Ornstein–Uhlenbeck process	Australia	Australian Journal of Agricultural and Resource Economics
46.	Sauter et al 2016	Indirectly considered Uncertainity of prices and costs	Market	Arithmetic Brownian motion	Norway	Journal of Forest Economics

Appendix S2: Continued

S.No	Paper	How they accounted for uncertainty	Uncertainty considered	Stochastic process (es)	Country	Journal
47.	Schatzki 2003	Returns from agriculture and forest as GBM	Market	Geometric Brownian motion	USA	Journal of Environmental Economics and Management
48.	Schiel et al 2018	Implicit consideration of Market uncertainty (prices volatility)	Market	Geometric Brownian motion	Germany	Journal of Business Economics
49.	Schou et al 2015	Subjective probability or perception of decision-maker about cc, they use Bayes rule to udpate beliefs	Climate change; perceptions	NI	Norway	Forest Policy and Economics
50.	Shahnazari et al 2014	Market uncertainity (electricity and carbon prices volatility) and political uncertainity (plociy jump arrival time)	Market, climate policy	MR and MA	Australia	Applied Energy
51.	Shahnazari et al 2017	Portfolio optimization under market and political uncertainty	Market	Geometric Brownian motion	Australia	Applied Energy
52.	Sisodia et al 2016	Market and regulatory uncertainty	Market	NI	Spain	Renewable Energy
53.	Song et al 2011	Return (price) uncertainty as GBM and MR	Market	Geometric Brownian motion & MR	USA	American Journal of Agricultural Economics
54.	Steinschneider et al 2012	Reasonal hydrolic forecasts of variability and cc, esemble of climate featues based of GCM (General Circulation Model)	Climate change	NI	USA	Water Resources Research
55.	Tee et al 2014	Carbon and timber prices as MR, Binomal tree	Market	Geometric Brownian motion & MR	New Zealand	Land Economics

Appendix S2: Continued

S.No	Paper	How they accounted for uncertainty	Uncertainty considered	Stochastic process (es)	Country	Journal
56.	Woodward et al 2011	considered high, medium and low emission senarios for UK	Climate change	NI	UK	Journal of Flood Risk Management
57.	Woodward et al 2014	considered high, medium and low emission senarios for UK focusing on sea level rise	Climate change	NI	UK	Risk Analysis
58.	Yemshanov et al 2015	Land values (= difference b/n crop price and cost as stochastic variable) as GBM	Market	Geometric Brownian motion	Canada	Forest Policy and Economics
59.	Zhu and Fan 2011	Technology uncertainty (volatility of ccs tech deployment cost) as controlled diffusion process, and thermal power generation cost uncertainty and carbon price as GBM	Market	Geometric Brownian motion	China	Applied Energy
60.	Zhu and Fan 2013	Electricity price as MR, carbon price and ccs operating cost as GBM	Market; climate policy	Geometric Brownian motion & MR	China	Energy
61.	Brown et al 2018	Uncertainities in sea level rises	Market		UK	Ocean and Coastal Management
62.	Kim et al 2018	Uncertainities in sea level rises (measured in several indicators)	Market		UK	Journal of Flood Risk Management
63.	Kontogianni et al 2014	Uncertainities in sea level rises	Market		Greece	Environmental science & policy
64.	Linquiti and Vonortas 2012	Uncertainities in sea level rises	Climate change		Indonesia	Climate Change Economics
65.	Oh et al 2018	Flood damage and real estate GDP volatilities	Climate change		Severall African countries	Journal of Cleaner Production

Appendix S2: Continued

S.No	Paper	How they accounted for uncertainty	Uncertainty considered	Stochastic process (es)	Country	Journal
66.	Srinivasan 2015	Project benefits (for the district)	Market		India	Mitig Adapt Strateg Glob Change
67.	Mense 2017	Level of amenity (general) as GBM	Environmental (pollution)	Geometric Brownian motion	Germany	Journal of Regional Science
